# Engineered Reporter Phages for Rapid Bioluminescence-Based Detection and Differentiation of Viable *Listeria* Cells

**DOI:** 10.1128/AEM.00442-20

**Published:** 2020-05-19

**Authors:** Susanne Meile, Anne Sarbach, Jiemin Du, Markus Schuppler, Carmen Saez, Martin J. Loessner, Samuel Kilcher

**Affiliations:** a Institute of Food, Nutrition, and Health, ETH Zurich, Zurich, Switzerland; University of Queensland

**Keywords:** bacteriophage, bioluminescence, CRISPR-Cas, food safety, *Listeria monocytogenes*, phage engineering, reporter phage, synthetic biology

## Abstract

The pathogen Listeria monocytogenes causes listeriosis, a severe foodborne disease associated with high mortality. Rapid and sensitive methods are required for specific detection of this pathogen during food production. Bioluminescence-based reporter bacteriophages are genetically engineered viruses that infect their host cells with high specificity and transduce a heterologous luciferase gene whose activity can be detected with high sensitivity to indicate the presence of viable target cells. Here, we use synthetic biology for *de novo* genome assembly and activation as well as CRISPR-Cas-assisted phage engineering to construct a set of reporter phages for the detection and differentiation of viable *Listeria* cells. Based on a single phage backbone, we compare the performance of four reporter phages that encode different crustacean, cnidarian, and bacterial luciferases. From this panel of reporter proteins, nanoluciferase (NLuc) was identified as a superior enzyme and was subsequently introduced into the genomes of a broad host range phage (A511) and two serovar 1/2- and serovar 4b/6a-specific *Listeria* phages (A006 and A500, respectively). The broad-range NLuc-based phage A511::*nluc*_CPS_ detects one CFU of L. monocytogenes in 25 g of artificially contaminated milk, cold cuts, and lettuce within less than 24 h. In addition, this reporter phage successfully detected *Listeria* spp. in potentially contaminated natural food samples without producing false-positive or false-negative results. Finally, A006::*nluc* and A500::*nluc* enable serovar-specific *Listeria* diagnostics. In conclusion, these NLuc-based reporter phages enable rapid, ultrasensitive detection and differentiation of viable *Listeria* cells using a simple protocol that is 72 h faster than culture-dependent approaches.

**IMPORTANCE** Culture-dependent methods are the gold standard for sensitive and specific detection of pathogenic bacteria within the food production chain. In contrast to molecular approaches, these methods detect viable cells, which is a key advantage for foods generated from heat-inactivated source material. However, culture-based diagnostics are typically much slower than molecular or proteomic strategies. Reporter phage assays combine the best of both worlds and allow for near online assessment of microbial safety because phage replication is extremely fast, highly target specific, and restricted to metabolically active host cells. In addition, reporter phage assays are inexpensive and do not require highly trained personnel, facilitating their on-site implementation. The reporter phages presented in this study not only allow for rapid detection but also enable an early estimation of the potential virulence of *Listeria* isolates from food production and processing sites.

## Abstract (precis)

Culture-dependent methods are the gold standard for sensitive and specific detection of pathogenic bacteria within the food production chain. In contrast to molecular approaches, these methods detect viable cells, which is a key advantage for foods generated from heat-inactivated source material. However, culture-based diagnostics are typically much slower than molecular or proteomic strategies. Reporter phage assays combine the best of both worlds and allow for near online assessment of microbial safety because phage replication is extremely fast, highly target specific, and restricted to metabolically active host cells. In addition, reporter phage assays are inexpensive and do not require highly trained personnel, facilitating their on-site implementation. The reporter phages presented in this study not only allow for rapid detection but also enable an early estimation of the potential virulence of *Listeria* isolates from food production and processing sites.

## INTRODUCTION

Listeria monocytogenes is an opportunistic, Gram-positive pathogen that can cause listeriosis, a severe foodborne disease associated with very high mortality rates (20 to 30%) among the young, old, pregnant, and immunocompromised (YOPI) risk group (1–3). Long incubation periods of invasive listeriosis of up to 67 days make it extremely difficult to track down sources of L. monocytogenes-associated outbreaks (4). In addition, *Listeria* grows at refrigeration temperatures, at low pH, and in the presence of high salt concentrations (5–7). Therefore, fast detection and early elimination of *Listeria* spp. from foods are high priorities and crucial for disease prevention. To this end, many countries enforce a zero-tolerance policy in ready-to-eat (RTE) foods, i.e., *Listeria* cells must not be present in 25 g of RTE food. To date, the gold standard for the detection of most foodborne bacterial pathogens is culture-based protocols (8). These methods are time-consuming and labor intensive (e.g., ISO 11290-1:2017 for *Listeria*). PCR-based, immunological, or mass spectrometry-based protocols are much faster but cannot differentiate between dead and viable cells, which is an important prerequisite when processed products are tested. In addition, these methods often require trained personnel and expensive machinery and are frequently compromised by food matrix effects, possibly leading to either false-positive or false-negative results (9).

Bacteriophages are viruses of bacteria that recognize and infect their host cells with unparalleled specificity and typically complete an infectious cycle in less than 1 h under appropriate growth conditions. Phage-encoded receptor-binding proteins mediate attachment and confer genus-, species-, or even serovar-level specificity (10, 11). Phages can be genetically engineered to encode reporter proteins that are produced upon infection, released from infected cells upon host lysis, and can be detected to indicate the presence of host bacteria (12, 13). A number of different reporter genes such as insect, bacterial, or crustacean luciferases, beta-galactosidase, fluorescent proteins, and the ice nucleation protein have previously been used to generate such diagnostic tools (14–21). Luciferase-based reporter phages are very sensitive and, following short preenrichments of less than 24 h in selective media, can detect cell numbers as low as 10 CFU/g of food (13).

Using an isogenic phage background, we present a systematic comparison of the suitability and performance of different luciferase systems for reporter phage construction. We identify nanoluciferase (NLuc) (22) as a superior reporter protein that enables direct detection of one to three *Listeria* cells under laboratory conditions. To highlight the potential of narrow host range reporter phages for diagnostic applications, we construct two *nluc*-based, serovar-specific phages that can be employed for rapid serovar differentiation of *Listeria* isolates. Finally, we construct a broad host range reporter phage based on *Listeria* phage A511, which is shown to reliably detect one single *Listeria* cell in 25 g of milk, chicken cold cuts, and iceberg lettuce in just 24 h, effectively shortening culture-dependent detection assay time by 72 h.

## RESULTS

### NLuc is a superior enzyme for the construction of reporter phages.

**FIG 1:**
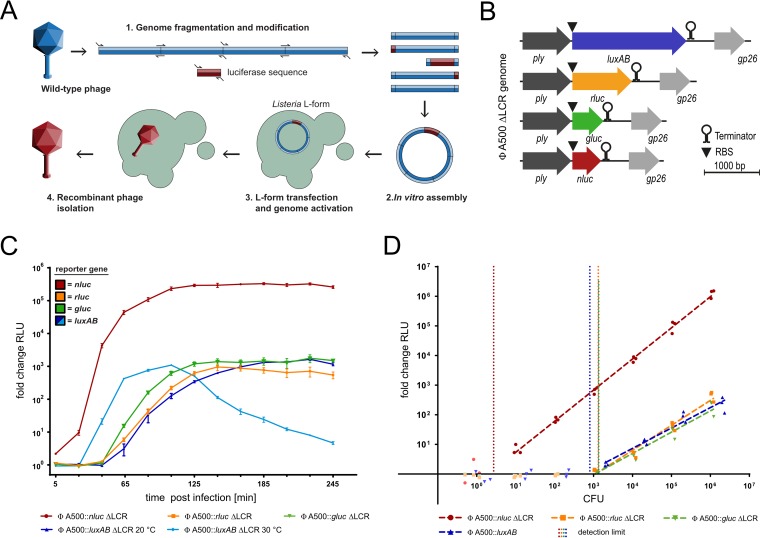
NLuc is a superior enzyme for the construction of synthetic and highly efficient *Listeria*-specific reporter bacteriophages. (A) Schematic representation of the synthetic phage engineering approach used to construct A500-derived reporter phages. (B) Comparison of the genetic location and size of the different luciferase genes integrated into the genome of A500 ΔLCR. (C) Bioluminescence time course assays of WSLC1042 cells infected with the indicated reporter phage at 30°C (unless indicated otherwise). (D) Reporter phage sensitivities were determined by infecting serial host cell dilutions and quantifying luminescence at 3 h p.i. Detection limits were calculated as the cell number required to produce a signal that is 3-fold above the standard deviation of the background luminescence (indicated as vertical dotted lines). All values are fold changes calculated by dividing the signal produced by A500::luciferase ΔLCR divided by the signal produced from infections with the parental phage lacking the luciferase gene (A500 ΔLCR). Ply, phage lysin; RLU, relative light unit. Data are mean ± standard error of the mean (SEM) from biological triplicates.

Luciferases are the most sensitive genetic reporters and have previously been used to construct a number of reporter phages that detect *Listeria*, *Mycobacterium*, *Bacillus*, *Yersinia*, *Escherichia*, *Salmonella*, *Erwinia*, and *Pseudomonas* species (16, 23–30). The choice of luciferase is likely to be an important determinant to consider when designing novel reporter phage candidates. Thus, we set out to construct and compare the performance of several isogenic reporter phages that carry different luciferase genes. As a genetic backbone, we selected the temperate siphovirus A500, which infects serovar (SV) 4b/6a *Listeria* strains. A500 features a 38.9-kb genome (31–33) and can be modified genetically using a recently developed synthetic phage engineering protocol (34). To this end, we assembled modified synthetic phage genomes *in vitro* from smaller overlapping DNA fragments (Tables S2 and S3) and subsequently activated them in L-form bacteria (Fig. 1A). To ensure a strictly lytic phenotype, we used a virulent derivative of A500 (A500 ΔLCR), which had previously been constructed to lack the entire lysogeny control region (LCR) (35). The A500 LCR (*gp31* to *gp33*) encodes both the integrase (*gp31*) and the repressor of the lytic cycle (*gp33*). Thus, A500 ΔLCR cannot establish or maintain lysogeny (35). For reporter phage construction, we selected four luciferases, derived from Vibrio harveyi (*luxAB*), Gaussia princeps (*gluc*), Renilla reniformis (*rluc*), and Oplophorus gracilirostris (*nluc*) (Promega). All nonbacterial luciferases were codon optimized (36) for expression in *Listeria* and synthesized *de novo* (Table S3). A strong ribosomal binding site (RBS) and the luciferase-coding sequences were inserted immediately downstream of the A500 endolysin gene (*ply*), which is highly expressed from a late viral promoter (Fig. 1B, Table S3). Synthetic phage genomes were assembled *in vitro* and activated in L-form bacteria, and the resulting reporter phages were isolated and verified by PCR and sequencing, and their plaque morphologies compared to the parental phage (Fig. S1B). To determine infection kinetics of the A500 ΔLCR-based reporter phages, we performed bioluminescence time course assays (see Materials and Methods) using infected L. monocytogenes SV 4b cultures (strain WSLC1042) (Fig. 1C). While there were luciferase-dependent differences, light emission from all reporter phages reached a plateau at around 2 to 3 h postinfection (p.i.). Due to instability of the fused LuxAB protein at elevated temperatures (23), infection by A500::*luxAB* ΔLCR must be carried out at 20°C to achieve a stable signal. At the plateau, the *nluc*-containing reporter phage A500::*nluc* ΔLCR reached >100-fold higher fold change (FC) values than all other reporter phages, indicating its superior light-emitting properties. The sensitivity of these reporter phage systems was subsequently quantified by infecting serial host cell dilutions and quantifying the FCs of light emission at 3 h p.i. (Fig. 1D). The detection limit is defined as the minimal number of bacteria required to produce a signal that is distinguishable from the background (i.e., background relative light unit [RLU] FC plus 3 times the standard deviation). Phage A500::*nluc* ΔLCR was able to directly detect as few as three WSLC1042 cells, while about 10^3^ CFU were needed to reliably detect L. monocytogenes using all other A500 ΔLCR-based reporter phage systems (detection limits were approximately 1,350, 1,320, and 800 cells for *gluc*, *rluc*, and *luxAB*, respectively). Thus, systematic comparison of different luciferases revealed the superior properties of NLuc for reporter phage construction. NLuc is a highly stable enzyme that produces strong background bioluminescence in crude phage lysates, which was not observed for any other tested luciferase. To remove residual NLuc protein after phage propagation and to obtain ultrapure phage preparations with minimal background luminescence, polyethylene glycol (PEG) precipitation and two rounds of CsCl density gradient ultracentrifugation were required (see Materials and Methods).

### CRISPR-Cas-assisted construction of broad host range *Listeria* reporter phages.

**FIG 2:**
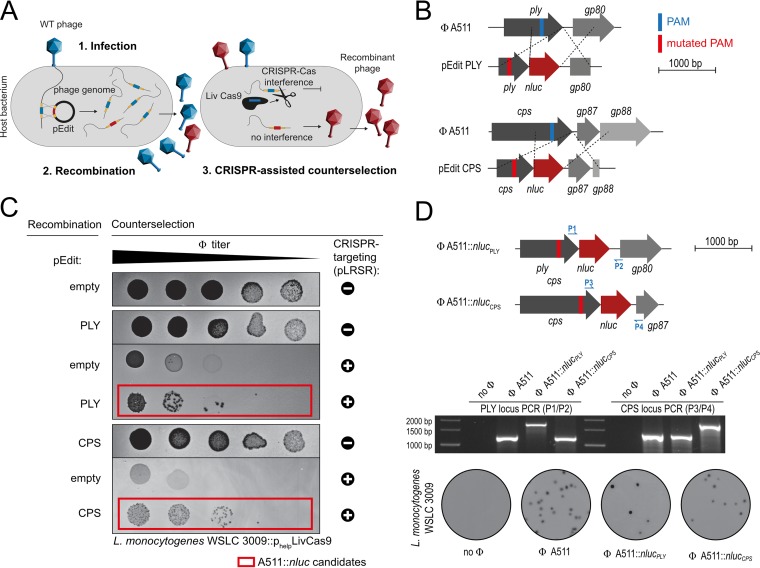
CRISPR-Cas9-aided construction of broad host range reporter phages A511::*nluc*_PLY_ and A511::*nluc*_CPS_. (A) Schematic representation of the two-step, CRISPR-Cas-assisted phage engineering approach using a programmable type II-A CRISPR-Cas system from L. ivanovii. Homologous recombination between the incoming A511 genome and an editing plasmid (pEdit) carrying the *nluc* sequence and flanking homology arms occurs at low frequency. The homology arms contain a protospacer sequence, which can be targeted by Cas9. However, a silent point mutation is introduced into the PAM motif of the pEdit homology arms, which allows for subsequent CRISPR-mediated, sequence-specific cleavage of nonmodified phage DNA, while recombinant genomes are protected and the corresponding phages enriched. (B) Schematic representation of the editing plasmids used to incorporate mutated PAMs and *nluc* gene sequences either downstream of the A511 major capsid protein (*cps*) or downstream of the A511 endolysin gene (*ply*). (C) Counterselection of wild-type phages. Serial dilutions of phages harvested after propagation on strains containing the indicated editing plasmids were spotted on L. ivanovii 3009 p_help_ *cas9* containing the indicated CRISPR targeting plasmids (crRNA encoding vector pLRSR). A511::*nluc* candidate phages that escape CRISPR-Cas interference are shown in red boxes. (D) Schematic representation of the resulting reporter phages A511::*nluc_PLY_* and A511::*nluc_CPS_*, including PCR-mediated genotype validation and assessment of plaque morphology. PAM, protospacer-adjacent motif; pLRSR, crRNA expression plasmids; p_help_, strong, constitutive *Listeria* promoter; pEdit empty, empty vector control without *nluc* or flanking homology arms; WT, wild type.

**FIG 3:**
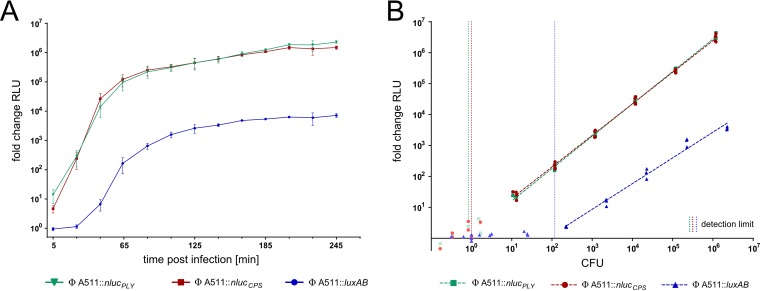
Ultrasensitive, A511-based NLuc reporter phages enable single-cell detection. The NLuc reporter phages A511::*nluc_PLY_* and A511::*nluc_CPS_* were benchmarked against the previously published phage A511::*luxAB*. All reporter phages featured comparable infection kinetics as determined by bioluminescence time course assays (A). However, dose-response curves demonstrate superior sensitivity of the NLuc-encoding phages, both of which are able to detect a single cell in this assay (B). Detection limits were calculated as the cell number required to produce a signal that is 3-fold above the standard deviation of the background luminescence (indicated as vertical dotted lines). All values are fold changes calculated by dividing the signal produced by A511::luciferase by the signal produced from infections with the parental wild-type phage. Data are mean ± SEM from biological triplicates.

Most *Listeria* phages feature narrow host ranges, which is a consequence of SV-specific host cell attachment. Only a few phages are known to infect a broad range of *Listeria* SVs and species, such as P100 and A511 (37, 38). In fact, A511 has been used for the construction of the first-generation *Listeria* reporter phage (A511::*luxAB*) (23, 39). To enable efficient and sensitive detection of all relevant *Listeria* SVs and species, we thus aimed at constructing an A511-based phage that transduces *nluc* as a reporter. Since the large 134-kb genome of A511 (38) cannot be assembled *in vitro*, we employed a homologous recombination-based and CRISPR-Cas-assisted counterselection approach (40) for construction of A511::*nluc* reporter phages. A workflow is shown in Fig. 2A. Briefly, A511 was propagated in the presence of a recombination template (editing plasmid), which carries the *nluc* gene as well as flanking homology regions to direct its sequence-specific integration. The resulting lysate constitutes a mixed-progeny population that contains both wild-type and recombinant phages. Subsequently, wild-type phages were actively counterselected using an L. ivanovii-derived type II-A CRISPR-Cas system (40), which was programmed to cleave wild-type but not recombinant genomes (Fig. 2A and B). Using this approach, we created two A511-derived reporter phages that carry the *nluc* gene either downstream of the A511 endolysin gene *ply* (A511::*nluc_PLY_*) or downstream of the major capsid gene *cps* (A511::*nluc_CPS_*) (Fig. 2C and D and Fig. S1C). Reporter phage-induced light emission was quantified from infected L. monocytogenes EGDe cells using time course bioluminescence assays and benchmarked against the previously published reporter phage A511::*luxAB*. Luminescence was detected as early as 5 min postinfection and reached a stable plateau with an RLU FC of 10^6^ at 180 min p.i., which is roughly 2 orders of magnitude brighter than the signal produced with A511::*luxAB* (Fig. 3A). Next, we determined the sensitivity of these reporter phage systems using dose-response assays in a growth medium. As previously published, A511::*luxAB* features a detection limit of 117 CFU (on strain EGDe), while both A511::*nluc*_CPS_ and A511::*nluc*_PLY_ are able to directly detect a single EGDe cell in this assay (Fig. 3B).

### Rapid serovar differentiation of *Listeria* spp. using narrow host range reporter phages.

**FIG 4:**
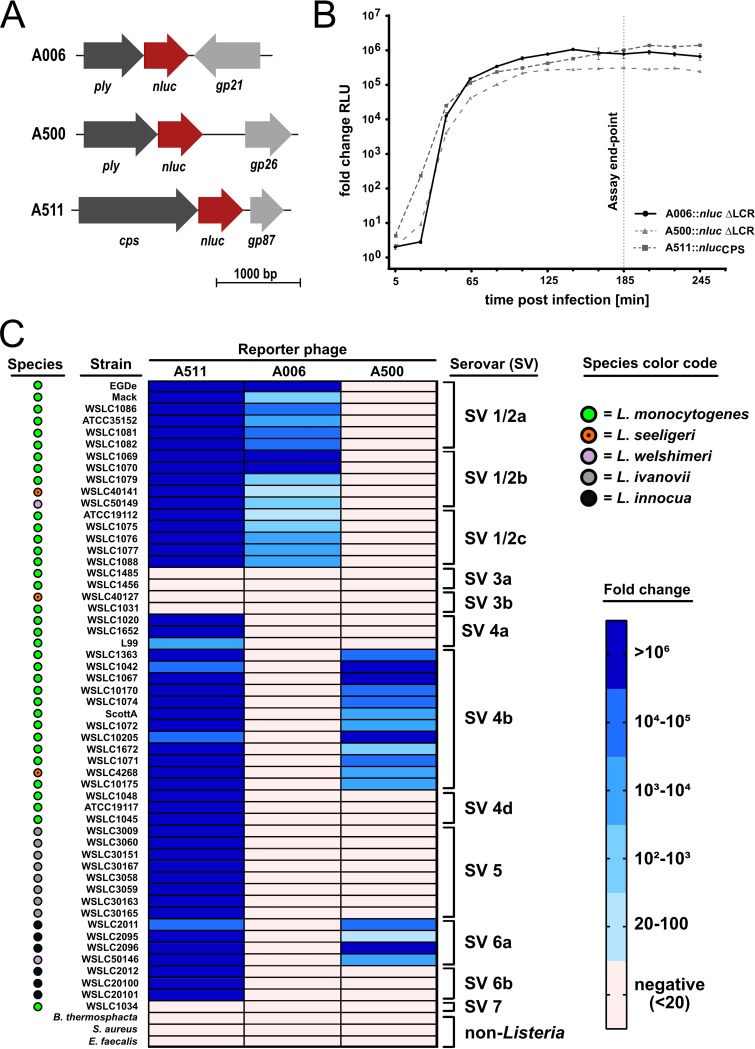
Serovar-specific reporter phages reliably differentiate *Listeria* strains. (A) Schematic representation of the luciferase gene insertion sites in the genomes of SV 1/2-specific phage A006::*nluc* ΔLCR, SV 4b/6a-specific phage A500::*nluc* ΔLCR, and broad host range phage A511::*nluc*_CPS_. (B) Time course luminescence assays were used to compare infection kinetics of phage A006::*nluc* ΔLCR (on EGDe) to those of phages A500::*nluc* ΔLCR (on WSLC1042) and A511::*nluc*_CPS_ (on WSLC3009). Three hours postinfection was selected as an optimal endpoint for bioluminescence assays with all reporter phages (vertical dotted line). Data are mean ± SEM from biological triplicates. (C) Reporter phage-mediated serovar differentiation of 54 *Listeria* strains covering the major SVs. Indicated strains were infected with reporter phages, and transduced bioluminescence was quantified at 3 h postinfection. Brothothrix thermosphacta, Staphylococcus aureus, and Enterococcus faecalis served as genus specificity controls. The heat map shows fold changes calculated by dividing the background-corrected signal produced by reporter phage infections by the signal produced from infections with the parental phage lacking the luciferase. ATCC, American Type Culture Collection; WSLC, *Listeria* strains from the Weihenstephan Microbial Strain Collection.

More than 90% of human L. monocytogenes infections are caused by SVs 1/2 and 4b (41, 42). While serovar differentiation is not a legal prerequisite for the assessment of food safety, it provides valuable information to estimate the potential virulence of food isolates. In addition, serotyping is an important tool for epidemiological outbreak investigations, including the tracking of sources of contamination within the production chain. *Listeria* serovars correlate with the architecture and specific glycosylation patters of wall teichoic acids (WTA), which also serve as primary phage receptors (43). This is why all temperate and most of the few virulent *Listeria* phages feature serovar-specific infection patterns. To enable rapid, phage-mediated differentiation of the two most relevant L. monocytogenes serovars, we generated an SV 1/2-specific NLuc reporter phage to complement our SV 4b/6a-specific phage A500::*nluc* ΔLCR. To this end, we first employed the L-form-assisted synthetic phage engineering platform (34) to delete the LCR (*gp21* to *gp25*) from the 38.1-kb temperate *Listeria* phage A006 (31) and subsequently introduced the *nluc* sequence downstream of the endogenous endolysin gene, yielding the SV 1/2-specific reporter phage A006::*nluc* ΔLCR (Fig. 4A). Genotypic and phenotypic characterization of this phage can be found in Fig. S1A. A time course luminescence assay of A006::*nluc* ΔLCR-infected L. monocytogenes EGDe cells was performed and compared to the SV4b/6a-specific reporter phage A500::*nluc* ΔLCR and the broad host range reporter phage A511::*nluc*_CPS_. For all reporter phages, bioluminescence reached a plateau at 3 h p.i., which was selected as a fixed endpoint for subsequent luminescence assays (Fig. 4B).

We tested reporter phage specificity on 54 *Listeria* strains covering 12 serovars (Fig. 4C), with a focus on SVs 1/2a, 1/2b, 1/2c, and 4b (28 strains). Target cells (10^7^ CFU/ml) were infected at a multiplicity of infection (MOI) of 1 and luminescence quantified at 3 h p.i. As previously described for A511 (33), A511::*nluc_CPS_* displayed a very broad host range and transduced all strains except for the rare SV groups 3 and 7. A006 ΔLCR::*nluc* was highly specific to the SV 1/2 group, whereas phage A500 ΔLCR::*nluc* transduces SV 4b and 6a strains. The SV cross-reactivity of A500 ΔLCR::*nluc* was expected because SVs 4b and 6a share an identical glycosylation pattern of the WTA ribitol-phosphate repeating unit, i.e., the same phage receptor epitope (43). Genus specificity was assessed on *Staphylococcus*, *Enterococcus*, and *Brochothrix* strains, the latter of which is a close phylogenetic relative of *Listeria* (44). None of the phages transduced any of these hosts. While *Listeria* phages are highly SV specific, they generally infect host cells independent of the species. Accordingly, we found that all phages were able to transduce other species, including L. seeligeri, L. welshimeri, L. ivanovii, and L. innocua, as long as the SV matched the phage host range (Fig. 4C). Interestingly, the SV-specific phages did not form plaques on many of the tested strains, but they were still able to transduce bioluminescence in an SV-specific fashion, i.e., inject DNA into these hosts (Fig. S2). In sum, we demonstrate that SV-specific *Listeria* reporter phages can be used to reliably and rapidly differentiate the most important *Listeria* SVs.

### A511::*nluc_CPS_* enables fast and highly sensitive detection of L. monocytogenes from foods.

**FIG 5:**
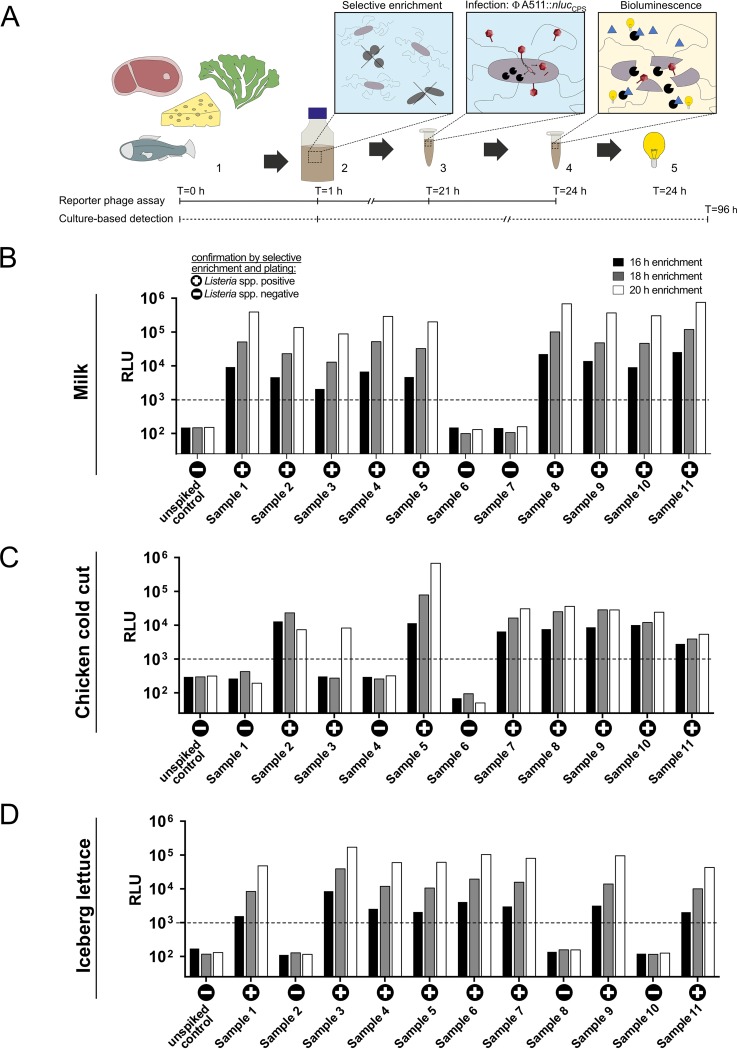
Bioluminescence-based detection of *Listeria* in artificially contaminated foods. (A) Schematic representation of the workflow and duration of the reporter phage-based detection assay compared to classical culture-dependent detection. (1) Preparation of the food sample; (2) *Listeria*-selective enrichment; (3) phage infection; (4) substrate addition; (5) detection of bioluminescence. (B to D) Bioluminescence output (RLUs) of artificially spiked food samples (samples 1 to 11) and unspiked controls is shown after selective enrichment in 1/4 LEB (16, 18, and 20 h) and compared to results from a culture-based detection approach performed with the same samples: +, culture positive for *Listeria*; −, culture negative for *Listeria*. Samples were contaminated with ± 1 CFU/25 g of food. Quantified values are 1.2 ± 0.2 in milk, 1.1 ± 0.04 in chicken cold cut (errors are SEM of biological duplicates), and 1.0 ± 0.2 in iceberg lettuce (error is standard deviation of technical triplicates). Dotted lines indicate threshold value.

The application of the broad host range reporter phage A511::*nluc_CPS_* was optimized for rapid and sensitive detection of L. monocytogenes in food (workflow is shown in Fig. 5A). To this end, samples of different RTE foods (milk, lettuce, and chicken cold cuts) were spiked with a single CFU of L. monocytogenes EGDe per 25 g of sample. Contaminated foods were subjected to selective enrichment in buffered *Listeria* enrichment broth (LEB) containing acriflavine, nalidixic acid, and cycloheximide at 4-fold diluted concentrations (1/4 LEB). Due to phage specificity, some growth of background flora is acceptable in this assay. An aliquot of the enriched cultures was removed after 16, 18, or 20 h and infected with A511::*nluc_CPS_*, and luminescence was quantified at 3 h p.i. For stochastic reasons, not all samples receive a single cell when using an inoculation as low as 1 CFU/25 g. Thus, we had to correlate our results with culture-based detection of the same samples (Fig. 5B to D, plus or minus). To this end, incubation of the remaining culture was continued until 48 h postinoculation in LEB, and the culture was subsequently grown on selective agar plates for an additional 48 h. We found that A511::*nluc_CPS_* produced a robust signal (>10^3^ RLUs) from all positive samples, and the reporter phage-derived results correlated 100% with the results obtained from culture-based detection (Fig. 5B). As expected, signals increased with longer preenrichment time. We propose that a 20-h preenrichment in 1/4 LEB produces the clearest results. We also tested these foods with preenrichment in full LEB (Fig. S3A to C, 5 samples each). While bioluminescence was generally slightly weaker, full LEB still produced 100% reliable results after 20 h of preenrichment. To address the performance of A511::*nluc_CPS_* in a difficult, high-fat food matrix, we applied the reporter phage protocol to artificially contaminated smoked salmon and found that the reporter phage reliably detected *Listeria* in this matrix as well, although a longer preenrichment (48 h) was required to produce reliable results (Fig. S3D).

In sum, we demonstrate that A511::*nluc_CPS_* enables reliable detection of 1 CFU of *Listeria* in contaminated food in as little as 24 h total assay time using a very simple protocol. Finally, we purchased 48 different food samples from local vendors and tested them for *Listeria* contamination using A511::*nluc_CPS_*. Our assay was benchmarked against the commercial 3M *Listeria* molecular detection assay as well as against standard selective enrichment in Fraser broth and plating (Aloa and Oxford agar, ISO 11290-1). To make all assays compatible, we used half Fraser broth instead of 1/4 LEB for initial liquid enrichment. Food samples included a variety of products ranging from salads, herbs, and sprouts to cheese, fish, and fruit (see Table S1). Four samples tested positive for *Listeria* in all assays (1× salmon, 3× salad), and the A511::*nluc_CPS_* reporter phage produced no false-positive or false-negative results. These data suggest that the reporter phage assay not only works in artificially spiked food samples but also reliably detects *Listeria* in real, potentially contaminated foods.

## DISCUSSION

Previously, the construction of recombinant phages has been a challenging and time-consuming task which typically relied on low-frequency homologous recombination and subsequent screening for recombinant phage clones (45, 46). Recently, more rapid and reliable phage genome engineering tools became available for *Listeria*, including CRISPR-Cas-assisted counterselection and L-form-assisted activation of synthetic DNA (34, 40). These approaches enable the systematic optimization of genome design principles as well as rapid construction and testing of genetically modified phages. For example, we optimized *Listeria* phage backbones for application as SV-specific diagnostic tools; *Listeria* phages with narrow SV specificity typically feature a temperate lifestyle and are therefore inherently unsuitable for reporter phage construction. To circumvent this restriction, we first reprogrammed the backbones of temperate phages A006 and A500 to become strictly lytic and subsequently engineered them as SV-specific reporter systems. In addition, we present the first systematic comparison of the performance of different luciferases in reporter phage assays, which demonstrate the superior sensitivity and signal stability of NLuc.

By using A006- and A500-based reporter phages, we were able to differentiate *Listeria* SVs 1/2 and 4b/6a, respectively. Although serotyping cannot be used to fully determine the *Listeria* species (and therefore pathogenicity), a positive signal with either of these two reporter phages indicates an L. monocytogenes contamination. This is because (i) 1/2 and 4b are the most prevalent L. monocytogenes SVs in food, and (ii) other food-associated *Listeria* species are either always (L. ivanovii, SV5) or predominantly (L. innocua and L. welshimeri, SVs 6a and 6b, respectively) associated with SVs other than 1/2 or 4b (47). Due to similarities in WTA architecture and glycosylation, our assay currently cannot differentiate SVs 4b and 6a, the latter of which is generally not associated with the L. monocytogenes species. Given that SV 1/2 and 4b L. monocytogenes strains cause the vast majority of documented cases of listeriosis (41, 42), our SV-specific reporter phages provide an early assessment of the potential virulence of L. monocytogenes food isolates. By using the set of broad and narrow host range reporter phages presented in this study, food producers can therefore rapidly detect *Listeria* spp. and obtain information about the pathogenicity and potential virulence of isolated strains. Although nonpathogenic *Listeria* species are not subject to the zero-tolerance policy, their presence is an important indicator of potential sources of contamination in the food production chain. In addition, other *Listeria* species may inhibit recovery of L. monocytogenes through competition during selective enrichment (48).

Akin to serotyping, *Listeria* phage-typing schemes allow for subdifferentiation of clinical isolates based primarily on WTA architecture (32, 43, 49). However, classical phage-typing approaches rely on plaque formation as a readout. Therefore, they require SV-specific phages that are able to complete an infectious cycle on most strains of a specific SV. Unfortunately, such phages do not exist for *Listeria*. We observed that the SV-specific NLuc reporter phages were able to transduce bioluminescence into all strains of their target SV, even if plaque formation could not be observed (see Fig. S2). Thus, NLuc reporter phages may be useful diagnostic tools to complement serological *Listeria* subdifferentiation schemes. In addition, such NLuc-encoding phages could also be used to study transduction and the effects of intracellular defense mechanisms on phage gene expression or as reporter systems to study and quantify phage promoter activity during infection.

Finally, we established an assay for NLuc-based detection of *Listeria* spp. in contaminated food products (Fig. 5, Table S1). We show that the A511::*nluc_CPS_* reporter phage assay meets the zero-tolerance policy requirements, i.e., reliably detects one cell in 25 g of spiked food. It does so with a total assay time of 24 h, which is 72 h faster than standardized selective plating methods such as ISO 11290-1. The ability of A511::*nluc_CPS_* to detect *Listeria* in commercial food samples (Table S1) suggests that this assay is reliable and independent of the growth or metabolic state of the initial contaminating cells. The only limitation so far is the inability of A511 to infect strains of the rare SV groups 3 and 7. It may be possible to adapt A511 to bind these strains or to isolate additional phage backbones with an even broader host range.

To avoid recalls of food products from distribution chains and consumers, diagnostic methods should ideally produce results within 1 day or less. For *Listeria* detection, such low assay times were recently achieved by combining specific capture and concentration of *Listeria* cells with subsequent reporter phage-assisted detection (50). Selective capture, separation, and concentration of *Listeria* cells were accomplished using cell wall-binding domains (CBDs) of phage endolysins that were coupled to paramagnetic beads. Higher costs associated with the functionalized bead technology and delicate handling steps, requiring automatic sampling and separation devices or skilled personnel, may prohibit on-site implementation of this technology. Here, we achieve similar assay times and ultrasensitive detection using a very simple and inexpensive assay.

In conclusion, we show how synthetic biology and CRISPR-Cas-assisted phage engineering allow for the construction of suitable *Listeria* reporter phages that reliably detect one CFU in 25 g of contaminated food in as little as 24 h. Our simple reporter phage assays are fully compatible with culture-dependent approaches and enable near online determination of *Listeria* contamination as well as an early assessment of the potential pathogenicity and virulence of strains isolated from food production and processing sites.

## MATERIALS AND METHODS

### Bacterial strains and growth conditions.

All L. monocytogenes and L. ivanovii strains were grown at 30°C in 0.5× brain heart infusion (BHI) (Biolife Italiana) broth. L. monocytogenes L-form strain Rev2L was grown in modified DM3 medium (5 g/liter tryptone, 5 g/liter yeast extract, 0.01% bovine serum albumin [BSA], 500 mM succinic acid, 5 g/liter sucrose, 20 mM K_2_HPO_4_, 11 mM KH_2_PO_4_, 20 mM MgCl_2_, pH 7.3) at 32°C (51). E. coli JM109 was grown in Luria-Bertani (LB) broth at 37°C.

### Phage propagation and purification and DNA isolation.

Phages were propagated using the soft agar overlay method (52). LC (LB agar, 0.4%, supplemented with 10 mM CaCl_2_, 10 mM MgSO_4_, and 10 g/liter glucose) was used as top agar, and 0.5× BHI served as the bottom agar layer. Phages were propagated on L. ivanovii WSLC3009 (A511) and L. monocytogenes WSLC1042 (A500) at 30°C or on L. monocytogenes EGDe (A006) at 25°C. Progeny virions were extracted with 5 ml SM buffer per plate (100 mM NaCl, 8 mM MgSO_4_, and 50 mM Tris, pH 7.4) and filter sterilized (0.2 μm) to obtain crude lysates. For DNA isolation, lysates were digested with DNase I (10 μg/ml) and RNase A (1 U/10 ml) at 37°C for 30 min, precipitated with polyethylene glycol (7% PEG 8000 and 1 M NaCl) in ice-water overnight, and pelleted by centrifugation (10,000 × *g* for 10 min at 4°C). Precipitated phages were digested with proteinase K (200 μg/ml at 55°C for 30 min in SM buffer supplemented with 10 mM EDTA, pH 8.0) and genomic DNA purified using the GenElute PCR clean-up kit (Sigma-Aldrich). For bioluminescence assays, reporter phages were purified from PEG-precipitated lysates by two rounds of CsCl density gradient ultracentrifugation (53) followed by two rounds of dialysis against 1,000× excess of SM buffer.

### Genome partitioning and assembly of synthetic genomes.

Genomes of bacteriophages were partitioned *in silico* into 4 to 6 fragments using primers designed to generate 40 nucleotide overlaps with adjacent fragments (Table S2). Genomic DNA or phage lysates were used as PCR templates (Table S2). The same approach was used to delete lysogeny control regions from the temperate phages A500 (35) and A006 (nucleotide [nt] positions 22809 to 25324). For reporter phage construction, the luciferase genes (*rluc*, *gluc*, and *nluc*) were codon optimized for expression in *Listeria* and generated by DNA synthesis (GeneArt string DNA fragments; Thermo Fisher) with a strong RBS (5′-GAGGAGGTAAATATAT-3′) upstream of the luciferase sequence (Table S3). The *luxAB* cassette was directly amplified from phage A511::*luxAB* (23). Primers and templates used for reporter gene insertion and phage genome amplification are listed in Table S2. Fragments were purified (GenElute PCR clean-up kit; Sigma-Aldrich) and assembled *in vitro* using Gibson one-step isothermal assembly (NEBuilder HiFi DNA assembly mastermix; New England BioLabs).

### L-form transfection and rebooting of phage genomes.

Synthetic phage genomes were reactivated as described previously (34). Briefly, cultures of L. monocytogenes L-form strain Rev2L were grown for 96 h in modified DM3 medium, and the optical density at 600 nm (OD_600_) was adjusted to 0.15. One hundred microliters of the L-forms was mixed thoroughly with 15 to 20 μl of synthetic phage DNA and 150 μl 40% PEG 20000 by pipetting. After 5 min of incubation at room temperature, 10 ml of prewarmed DM3 medium was added, and the mixture was incubated at 32°C for 24 h without agitation. Fifty to 500 μl of the transfected L-form cultures was mixed with 200 μl of the bacterial host strains in 5 ml of LC soft agar and poured onto 0.5× BHI bottom agar, followed by overnight incubation at 30°C to allow for plaque formation. After three rounds of plaque purification, phages were expanded until semiconfluent lysis was achieved. Correct insertion of reporter gene sequences was verified by PCR and Sanger sequencing (Table S2).

### Construction of A511::*nluc*_PLY_ and A511::*nluc*_CPS_ reporter phages.

Two editing plasmids were constructed to mediate integration of the codon-optimized *nluc* gene sequence and a strong ribosomal binding site (5′-GAGGAGGTAAATATAT-3′) downstream of the A511 endolysin gene (*ply511*) and major capsid gene (*cps511*). The homology arms flanking the *nluc* gene contained silent mutations in the protospacer-adjacent motif (PAM) that allow for CRISPR escape of recombinant phage genomes. Plasmids were constructed by Gibson isothermal assembly using pSK1_cps511_lys-his6 or pSK1_ply511_lys-his6 as backbones (40) and the synthetic, codon-optimized *nluc* sequence as an insert. The resulting editing templates pEdit CPS (full name, pSK1*_cps511_nluc*) and pEdit PLY (pSK1*_ply511_nluc*) were amplified in E. coli JM109 and transformed into L. ivanovii WSLC3009. Primers and templates used for plasmid construction are listed in Table S2. Subsequently, pEdit-containing strains were infected with phage A511 using soft agar overlays to obtain semiconfluent lysis, and the resulting mixtures containing wild-type and recombinant phages were extracted and subjected to counterselection on corresponding LivCRISPR-1 Cas9 and CRISPR RNA (crRNA)-expressing strains (WSLC3009::Phelp_Cas9 pLRSR_A511ply or WSLC3009::Phelp_Cas9 pLRSR_A511cps) (40). Several plaques were isolated for each phage, and the correct genotype was assessed by PCR and Sanger sequencing (Table S2).

### Bioluminescence time course assay.

Stationary-phase cultures of *Listeria* were diluted to an OD_600_ of 0.01 using 0.5× BHI. The cultures were infected with 1 × 10^7^ PFU/ml phage and incubated at 30°C or 20°C at 150 rpm (Table S4). Bioluminescence was quantified (integration time, 5 s; delay, 2 s) every 20 min for 240 min in white, flat-bottom 96-well plates (uncoated PP, Nunc; Thermo Fisher) using a luminometer (GloMax navigator; Promega) with an injector for substrate addition (0.35% nonanal in 70% EtOH; Renilla luciferase assay system; Promega) according to Table S4. To reduce the signal-to-noise ratio, the NLuc substrate (Nano-Glo luciferase assay system; Promega) was added 5 min prior to measurement according to the manufacturers’ instructions. Relative light units (RLUs) were background corrected by subtraction of luminescence values obtained from phage-only controls. RLU FCs were calculated by dividing background-corrected luminescence values from infections with reporter phages by values obtained from infections with isogenic control phages that do not encode a luciferase.

### Sensitivity and dose-response assays.

Ten-fold dilution series ranging from OD_600_ 10^−1^ to 10^−9^ were prepared from stationary-phase *Listeria* cultures in 0.5× BHI medium and infected with 10^7^ PFU/ml phage. Bacterial counts of the dilution series were determined by plating on 0.5× BHI agar prior to infection. Infected cultures were incubated, and bioluminescence was measured according to Table S4. Fold changes of background-corrected RLUs (RLU reporter phage/RLU isogenic control phage) were calculated and plotted against the CFU of the corresponding host dilutions. To calculate the minimum of CFU detectable by the reporter systems (detection limit), a linear regression curve was fitted through log_10_ values of those calculated data points that were above the background luminescence. The intersection of the curve with the 3-fold standard deviation of the background signal was defined as the detection limit of the system.

### *Listeria* serovar differentiation.

*Listeria* cultures were diluted in 0.5× BHI supplemented with 1 mM CaCl to an OD_600_ of 0.01 and infected with reporter phages A006::*nluc* ΔLCR, A500::*nluc* ΔLCR, and A511::*nluc_CPS_* and corresponding isogenic, luciferase-deficient control phages at an MOI of 1. Bioluminescence was quantified after 180 min of infection at 30°C according to Table S4. FCs of background-corrected RLUs (RLU reporter phage/RLU isogenic control phage) were calculated. To test the plaquing ability of the phages, 10-μl spots of serially diluted phages A006::*nluc* ΔLCR, A500::*nluc* ΔLCR, and A511::*nluc_CPS_* were spotted on plates (LC top agar, 0.5× BHI bottom agar) whose top agar layers had previously been inoculated with stationary-phase cultures of indicated host strains.

### *Listeria* detection in spiked food samples.

Iceberg lettuce, chicken cold cuts, and pasteurized whole milk were obtained at local grocery stores. We prepared 1-cm^2^ slices of food products and divided them into portions of 25 g in blender bags. Individual samples were spiked with 1 CFU of a stationary-phase culture of L. monocytogenes EGDe. For each food matrix, one unspiked sample served as a negative control. The correct CFU input was confirmed by plating on 0.5× BHI. Samples were homogenized either with 100 ml buffered LEB (Fig. S1A to C) (36.1 g/liter *Listeria* enrichment broth [Biolife Italiana], 28 mM K_2_HPO_4_, 5 mM KH_2_PO_4_) or with 100 ml 1/4 LEB (Fig. 5B to D, Fig. S3D) (9.1 g/liter *Listeria* enrichment broth, 28 mM K_2_HPO_4_, 5 mM KH_2_PO_4_, 30 g/liter tryptic soy broth [Biolife Italiana], 4.5 g/liter yeast extract [Biolife Italiana]) containing the same amount of nutrients and buffering agents but fewer antibiotics. The blender bags were sealed and incubated at 30°C at 155 rpm for selective enrichment of *Listeria* spp. After 16, 18, and 20 h, aliquots of the enriched samples were removed and infected with 10^7^ PFU/ml of phage A511::*nluc*_CPS_. At 3 h p.i., luminescence measurements were carried out as described above with 100 μl sample and 100 μl substrate. To monitor *Listeria* growth, enriched samples were streaked on Oxford agar (Biolife Italiana) at each time point and incubated at 37°C for 48 h. After 48 h of selective enrichment in the blender bags, all samples were streaked on Oxford and Aloa agar (Biolife Italiana) and incubated for another 48 h at 37°C. The presence of L. monocytogenes on the plates was assessed by colony morphology and microscopy, and results were compared to bioluminescence measurements.

### *Listeria* detection in food.

Twenty-five grams of food was mixed with 100 ml half-Fraser bouillon in blender bags, homogenized, and incubated for 20 h (reporter phage assay) to 24 h (molecular detection and selective plating) at 30°C. Ferric ammonium citrate was omitted from the half-Fraser recipe because this compound interferes with luminescence measurements, and reporter phage assays were performed in this broth as described above. At 24 h postenrichment, molecular *Listeria* detection was done according to the manufacturer’s instructions (3M *Listeria* molecular detection assay; catalog no. MDA2LIS96). In addition, preenriched cultures were plated on Aloa and Oxford agar for an additional 48 h. Potential conflicting results were resolved by microscopy (exclude cocci and spore formers) or 16S sequencing. API tests (API Listeria, bioMérieux) were performed to identify the *Listeria* species from positive food isolates.

## Supplementary Material

Supplemental file 1
