# Supplementary material for: Engineered Reporter Phages for Rapid Bioluminescence-Based Detection and Differentiation of Viable Listeria Cells
Source: Appl Environ Microbiol. 2020 May 19;86(11):e00442-20. doi: 10.1128/AEM.00442-20 (PMC7237785; doi:10.1128/AEM.00442-20)
Supplement: Supplemental file 1 [file AEM.00442-20-s0001.pdf]

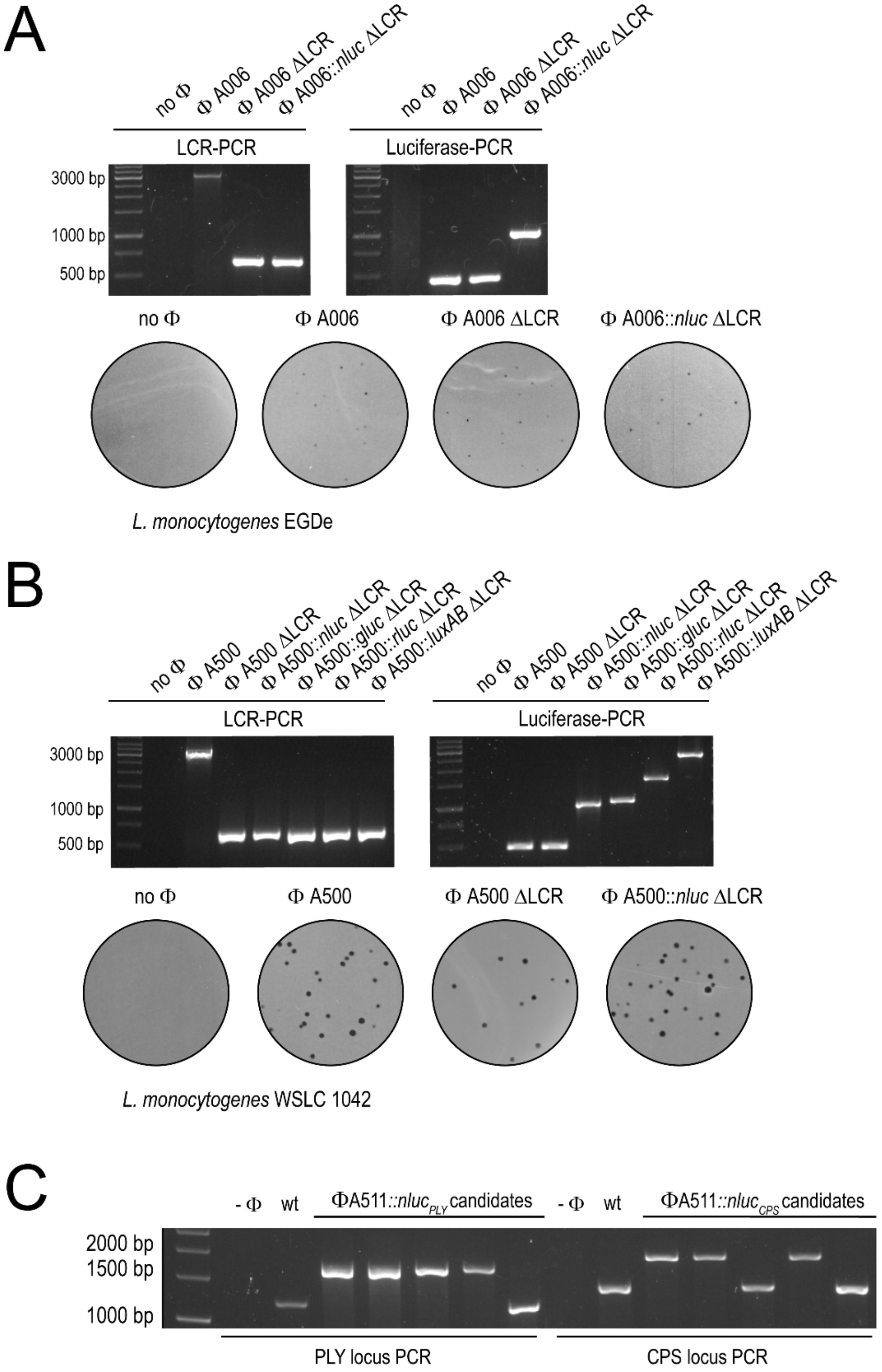

Supplementary Figure S1: Genotypic and phenotypic analysis of engineered phages used in this study. PCR verification and plaque morphologies of A006-derived phages (**A**) and A500-derived phages (**B**) are shown. Deletions of the lysogeny control regions (LCR-PCR) and correct insertion of luciferase sequences (Luciferase-PCR) were verified by PCR and plaque morphologies visualized from soft-agar overlays after 24 h of incubation. (**C**) Upon CRISPR-Cas-mediated counterselection, five individual plaques were picked and insertion of the heterologous *nluc* gene sequence into the *ply*- or *cps*-locus of A511-derived candidates was verified by PCR ("PLY locus PCR" and "CPS locus PCR", respectively). Primers are listed in Table S1.

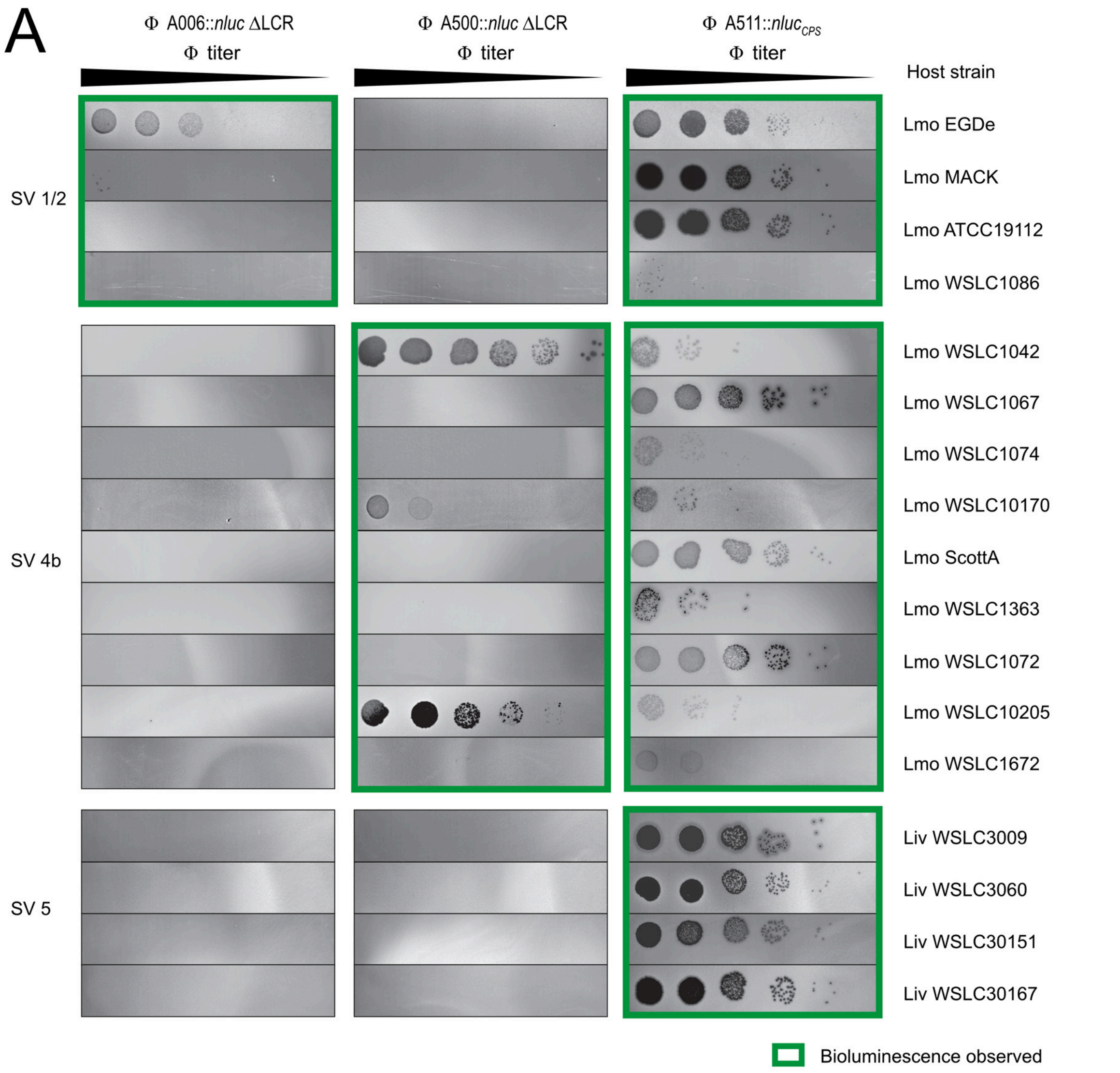

Supplementary Figure S2: Phagetyping and SV differentiation of *Listeria* spp. by bioluminescence measurements and spot-on-the-lawn plaque assays. (**A**) Indicated strains covering *Listeria* SVs 1/2, 4b, and 5 were infected with serial dilutions of the indicated phage using spot-on-the-lawn assays and plaque formation was visualized 24 h p.i. Host strains that produced a positive signal with the indicated phages in the bioluminescence assays are highlighted within green boxes.

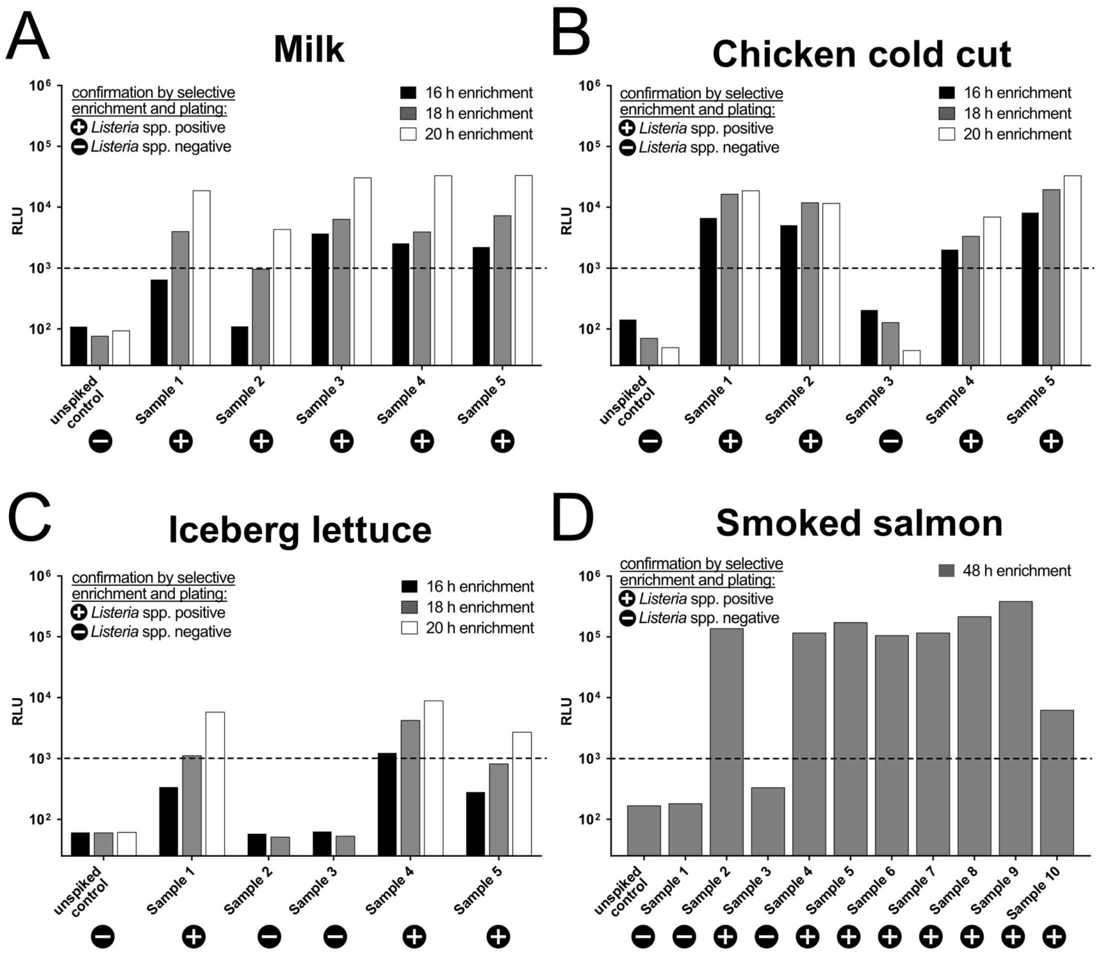

Supplementary Figure S3: Bioluminescence-based detection of *Listeria* in artificially contaminated foods. Bioluminescence (RLU) of artificially spiked milk (A), chicken cold cut (B), and iceberg lettuce (C) are shown after selective enrichment in full LEB (16, 18, and 20 h) and compared to results from culture-based detection of the same samples. Spiked salmon samples (D) were enriched for 48 h in 1/4 LEB. For each food matrix, one unspiked control was included. (+) culture positive for *Listeria*; (-) culture negative for *Listeria*. Samples were contaminated with ± 1 CFU / 25 g of food (quantified values: 1.4 ±0.3 in milk, 1.1 ± 0.1 in chicken cold cut, 1.4 ± 0.1 in iceberg lettuce and 1.0 ± 0.2 in smoked salmon, errors are STDEV of technical triplicates). Dotted lines = threshold for positive samples.

Supplementary Table S1. *Listeria* detection in potentially contaminated food samples from commercial sources. Exclusion = experimental clarification of unclear selective plating results; - = no growth or negative results; + = growth or positive results; +/- = some growth, untypical morphology; *C. citreum* = *Curtobacterium citreum.*

**Food sample**	**3M MDS**	**Selective plating**		**Reporter phage**		**Exclusion**	**API test**
(anonym. vendor)		Oxford	ALOA	Result	FC-value		API® Listeria
Pineapple pieces	-	-	-	-	1.42	nA	
Salad with beans and pomegranate	-	-	-	-	2.15	nA	
Alfa-alfa sprouts	-	-	-	-	1.20	nA	
Cheese "Mostkäse"	-	-	-	-	1.80	nA	
Salad with carots, shaker	-	-	-	-	1.50	nA	
Salad, organic	-	-	-	-	0.05	nA	
Spicy thai basil	-	-	-	-	1.36	nA	
Cress	-	-	-	-	0.8	nA	
**Mixed salad, organic**	**+**	**+**	**+**	**+**	**172222.22**	**nA**	***L.grayi***
Onion sprouts	-	-	-	-	2.00	nA	
Rocket sprouts	-	-	-	-	2.09	nA	
Sprouts	-	-	-	-	1.65	nA	
**Salmon**	**+**	**+**	**+**	**+**	**55.71**	**nA**	***L.welshimeri***
Thai coriander	-	-	-	-	3.41	nA	
Greek salad	-	-	-	-	3.43	nA	
Mungbean sprouts, organic	-	-	-	-	3.41	nA	
Salad, organic	-	-	-	-	3.67	nA	
Coriander	-	-	-	-	3.39	nA	
Mixed salad	-	-	+/-	-	1.50	16S sequence (*C. citreum*)	
**Salad**	**+**	**+**	**+**	**+**	**8150.00**	**nA**	***L.welshimeri***
Microgreens	-	-	-	-	3.89	nA	
Mungbean sprouts	-	-	-	-	4.95	nA	
Mixed sprouts	-	-	-	-	1.70	nA	
Sweet basil	-	-	-	-	1.77	nA	
Mixed salad	-	-	+/-	-	1.49	Spore-forming	
Mixed sprouts	-	-	-	-	1.90	nA	
Cheese and ham salad	-	-	-	-	3.32	nA	
Thai coriander	-	-	-	-	2.00	nA	
Parsley	-	-	-	-	4.90	nA	
Salad	-	-	-	-	3.75	nA	
Thai celery	-	-	-	-	2.33	nA	
Basil	-	-	-	-	2.19	nA	
Rice sandwich with smoked salmon	-	-	-	-	1.67	nA	
Filled pimiento	-	-	-	-	2.30	nA	
Coriander	-	-	-	-	2.79	nA	
Thai basil	-	-	-	-	2.43	nA	
**Salad, organic**	**+**	**+**	**+**	**+**	**19500.00**	**nA**	***L innocua***
Fleur-de-lis sprouts	-	-	-	-	1.51	nA	
Smoked salmon	-	-	-	-	2.22	nA	
Cicorino rosso	-	-	-	-	1.30	nA	
Chicken salad	-	-	-	-	1.93	nA	
Vietnamese coriander	-	-	-	-	2.17	nA	
Mixed sprouts (alfa-alfa and rocket)	-	-	+/-	-	1.98	Spore-forming	
Crayfish salad	-	-	-	-	1.52	nA	
Mixed sprouts	-	-	-	-	2.08	nA	
Salad	-	-	+/-	-	1.02	Spore-forming	
Smoked salmon with herbs	-	-	-	-	1.84	nA	
Egg and chicken salad	-	-	-	-	1.49	nA	

Supplementary Table S2. Primers and templates used for phage genome fragmentation, modification, PCR-based insert validations, and sequencing of phages and plasmids.

Supplementary Table S2: Primers and templates used for phage genome fragmentation, modification, PCR-based insert validations, and sequencing of phages and plasmids.

	Fragment ID	Template	Primer 1	P1 sequence [5'-3']	Primer 2	P2 sequence [5'-3']	Amplicon size [bp]
ΦA500::*rluc* ΔLCR assembly	A500 f1	A500 gDNA	A500 f1 fwd	ATGCTACAGAAGCCGCTCGTC	A500 f1 rev	CATCACCTAAACCGACTCTAAACAGTT	8492
	A500 luc f2	A500 ΔLCR gDNA	A500 f2 fwd	CAGCTTGGCACGGAACTGTTTAGA	A500 luc f2 rev	ATATATATTTACCTCCTCTTATTTTAAGAAGTATTCTGCTGTGTAATACC	12373
	A500 rluc f3A	rluc string	A500 rluc f3A fwd	CAGAATACTTCTTAAAATAAGAGGAGGTAAATATATATGGGTGGTC	A500 rluc f3A rev	AAAATGCGAGGGCAATAGTTTTATTGTTCGTTTTTTAAAACACGTTCAACGAAAG	1112
	A500 rluc f3B	A500 ΔLCR gDNA	A500 rluc f3B fwd	TTTTAAAAAACGAACAATAAAACTATTGCCCTCGCATTTTGCG	A500 f3 rev	AAGTGTCTTGTAACGTTTCTGTGTCTA	9138
	A500 f4	A500 gDNA	A500 f4 fwd	ATGGCGGAGGACTTAGACACAGA	A500 f4 rev	TTCGAACTATAACCAGCAAGACGAG	9016
ΦA500::*gluc* ΔLCR assembly	A500 f1	A500 gDNA	A500 f1 fwd	ATGCTACAGAAGCCGCTCGTC	A500 f1 rev	CATCACCTAAACCGACTCTAAACAGTT	8492
	A500 luc f2	A500 ΔLCR gDNA	A500 f2 fwd	CAGCTTGGCACGGAACTGTTTAGA	A500 luc f2 rev	ATATATATTTACCTCCTCTTATTTTAAGAAGTATTCTGCTGTGTAATACC	12373
	A500 gluc f3A	gluc string	A500 gluc f3A fwd	CAGAATACTTCTTAAAATAAGAGGAGGTAAATATATATGGGTGTTAAAGT	A500 gluc f3A rev	AAAATGCGAGGGCAATAGTTTTAATCACCACCAGCACCTTTGATTTTA	614
	A500 gluc f3B	A500 ΔLCR gDNA	A500 gluc f3B fwd	AAGGTGCTGGTGGTGATTAAAACTATTGCCCTCGCATTTTGCG	A500 f3 rev	AAGTGTCTTGTAACGTTTCTGTGTCTA	6566
	A500 f4	A500 gDNA	A500 f4 fwd	ATGGCGGAGGACTTAGACACAGA	A500 f4 rev	TTCGAACTATAACCAGCAAGACGAG	9016
ΦA500::*nluc* ΔLCR assembly	A500 f1	A500 gDNA	A500 f1 fwd	ATGCTACAGAAGCCGCTCGTC	A500 f1 rev	CATCACCTAAACCGACTCTAAACAGTT	8492
	A500 luc f2	A500 ΔLCR gDNA	A500 f2 fwd	CAGCTTGGCACGGAACTGTTTAGA	A500 luc f2 rev	ATATATATTTACCTCCTCTTATTTTAAGAAGTATTCTGCTGTGTAATACC	12373
	A500 nluc f3A	nluc string	A500 nluc f3A fwd	CAGAATACTTCTTAAAATAAGAGGAGGTAAATATATATGGTTTTCACTT	A500 nluc f3A rev	AAAATGCGAGGGCAATAGTTTTAAGCTAAGATACGTTCACATAAACGC	572
	A500 nluc f3B	A500 ΔLCR gDNA	A500 nluc f3B fwd	GTGAACGTATCTTAGCTTAAAACTATTGCCCTCGCATTTTGCG	A500 f3 rev	AAGTGTCTTGTAACGTTTCTGTGTCTA	6566
	A500 f4	A500 gDNA	A500 f4 fwd	ATGGCGGAGGACTTAGACACAGA	A500 f4 rev	TTCGAACTATAACCAGCAAGACGAG	9016
ΦA500::*luxAB* ΔLCR assembly	A500 f1	A500 gDNA	A500 f1 fwd	ATGCTACAGAAGCCGCTCGTC	A500 f1 rev	CATCACCTAAACCGACTCTAAACAGTT	8492
	A500 luc f2	A500 ΔLCR gDNA	A500 f2 fwd	CAGCTTGGCACGGAACTGTTTAGA	A500 luc f2 rev	ATATATATTTACCTCCTCTTATTTTAAGAAGTATTCTGCTGTGTAATACC	12373
	A500 luxAB f3A	A511::*luxAB*	A500 luxAB f3A fwd	CAGAATACTTCTTAAAATAAG*AGGAGG*TAAATATATATGAAATTTGG	A500 luxAB f3A rev	AAAATGCGAGGGCAATAGTTTTACGAGTGGTATTTGACGATGTTG	2099
	A500 luxAB f3B	nluc string	A500 luxAB f3B fwd	TCGTCAAATACCACTCGTAAAACTATTGCCCTCGCATTTTGCG	A500 f3 rev	AAGTGTCTTGTAACGTTTCTGTGTCTA	6566
	A500 f4	A500 gDNA	A500 f4 fwd	ATGGCGGAGGACTTAGACACAGA	A500 f4 rev	TTCGAACTATAACCAGCAAGACGAG	9016
ΦA006 ΔLCR assembly	A006 f1	A006 gDNA	A006 f1 fwd	TTCAGATAAGACAATGCCACTTGTG	A006 f1 rev	GAATATCCTAGCGAATGCGAAATAG	9985
	A006 f2	A006 gDNA	A006 f2 fwd	TGAACTATGTCGGTCCTATTTCG	A006 f2 rev	AATAAATACAATACTTACACCTGGAATG	9833
	A006 DLCR f3A	A006 gDNA	A006 ΔLCR f3A fwd	ATTACAATAGGCCATTCCAGGTG	A006 ΔLCR f3A rev	AATATGAAAGTCAATGAAATAGTTAATGGACAAGATTAATGAAATTTAAAAC	3549
	A006 DLCR f3B	A006 gDNA	A006 ΔLCR f3B fwd	ATTAATCTTGTCCATTAACTATTTCATTGACTTTCATATTTACCACC	A006 ΔLCR f3B rev	CCAAAGATCATCAACAACCATCG	3686
	A006 f4	A006 gDNA	A006 f4 fwd	AAACATGGTTGAGAATCCGATGG	A006 f4 rev	CTTACTGGCAATTTTCACAAGTGG	9294
ΦA006::*nluc* ΔLCR assembly	A006 f1	A006 gDNA	A006 f1 fwd	TTCAGATAAGACAATGCCACTTGTG	A006 f1 rev	GAATATCCTAGCGAATGCGAAATAG	9985
	A006 nluc f2A	A006 gDNA	A006 nluc f2A fwd	TGAACTATGTCGGTCCTATTTCG	A006 nluc f2A rev	CCATATATATTTACCTCCTCCTAAATCTTTTTAACAAACTTCTTGTTAGC	9186
	A006 nluc f2B	nluc string	A006 nluc f2B fwd	TTTGTTAAAAAGATTTAGGAGGAGGTAAATATATATGGTTTTCACTTTAG	A006 nluc f2B rev	CGCGAGGGCTTTAAACTAAATCTTAAGCTAAGATACGTTCACATAAACG	572
	A006 nluc f3	A006 ΔLCR gDNA	A006 nluc f2B fwd	GAACGTATCTTAGCTTAAGATTTAGTTTAAAGCCCTCG	A006 f4 rev	CCAAAGATCATCAACAACCATCG	7308
	A006 f4	A006 gDNA	A006 f4 fwd	AAACATGGTTGAGAATCCGATGG	A006 f4 rev	CTTACTGGCAATTTTCACAAGTGG	9294
pSK1_*ply511_nluc*	pSK1 PLY backbone	pSK1_*ply511_lys-his6*	pSK1 ply511 fwd	TATCTTAGCTTAAATAATGGTAAACTAGAGGAGACTTT	pSK1 ply511 rev	TATATATTTACCTCCTCTTATTTTTTGATAACTGC	5169
	nluc *ply511*	nluc string	ply511 nluc fwd	AGTTATCAAAAAATAAGAGGAGGTAAATATATATGGTTTTCAC	ply511 nluc rev	TCTAGTTTACCATTATTTAAGCTAAGATACGTTCACATAAACG	564
pSK1_*cps511_nluc*	pSK1 CPS backbone	pSK1_*cps511_lys-his6*	pSK1 cps511 fwd	CGTATCTTAGCTTAATAATTATAGGATAATTGAATAAAAACAGTATAGAG	pSK1 ply511 rev	CATATATATTTACCTCCTCTTAGTTGC	5371
	nluc *cps511*	nluc string	cps511 nluc fwd	TTCATAGCAACTAAGAGGAGGTAAATATATATGGTTTTCAC	cps511 nluc rev	AATTATCCTATAATTATTAAGCTAAGATACGTTCACATAAACG	562
A500 Luciferase PCR, sequencing	A500	A500	A500 LCR fwd	AGCTTTTGCGATGTTGTAGC	A500 LCR rev	TATCATAACTAATCCCACAACC	484
	A500 ΔLCR	A500 ΔLCR					484
	A500 rluc	A500::*rluc* ΔLCR					1557
	A500 nluc	A500::*nluc* ΔLCR					1016
	A500 gluc	A500::*gluc* ΔLCR					1059
	A500 luxAB	A500::*luxAB* ΔLCR					2544
A500 LCR PCR	A500	A500	A500 luc fwd	CATAAACCGCGTAACAAAGCC	A500 luc fwd	GTTTTTCATTTTCCACCCCTC	3191
	A500 ΔLCR	A500 ΔLCR					619
	A500 rluc	A500::*rluc* ΔLCR					619
	A500 nluc	A500::*nluc* ΔLCR					619
	A500 gluc	A500::*gluc* ΔLCR					619
	A500 luxAB	A500::*luxAB* ΔLCR					619
A006 Luciferase PCR, sequencing	A006	A006	A006 luc fwd	CCATCGACAAGTGCAAGCAA	A006 luc rev	GGTAAAGATACTCTTCCTCTAGCCA	451
	A006 ΔLCR	A006 ΔLCR					451
	A006 nluc	A006::*nluc* ΔLCR					990
A006 LCR PCR, sequencing	A006	A006	A006 LCR fwd	CCTGGTAATAATCAAGGCTAGTTTATGTTATAGTA	A006 LCR rev	ATATATTGCATTTTAGTATCTTTGCCAAAAGAGGC	3197
	A006 ΔLCR	A006 ΔLCR					681
	A006 nluc	A006::*nluc* ΔLCR					681
A511 PLY locus PCR	A511	A511	A511 PLY fwd	TAAGCTAGGAGGAACAACAC	A511 PLY fwd	TTGCTTTCTGCCGTTTTAGC	1177
	A511 nluc PLY	A511::*nluc*$_{PLY}$					1709
	A511 nluc CPS	A511::*nluc*$_{CPS}$					1177
A511 CPS locus PCR	A511	A511	A511 CPS fwd	AACTTGTTCGCGATAACGG	A511 CPS fwd	TATGATTGTAGAAGGTGTCTC	1346
	A511 nluc PLY	A511::*nluc*$_{PLY}$					1346
	A511 nluc CPS	A511::*nluc*$_{CPS}$					1912
Sequencing pEdit PLY	n/a	pSK1_*ply511_nluc*	pSK1 fwd	GAGAAATGGAAGTTGAATTAAG	n/a	n/a	n/a
	n/a		pSK1 rev	TTTATACTGCAATCGGATG	n/a	n/a	n/a
	n/a		A511 PLY fwd-2	AGAGGAAACGGTGTATACG	n/a	n/a	n/a
Sequencing pEdit CPS	n/a	pSK1_*cps511_nluc*	pSK1 fwd	GAGAAATGGAAGTTGAATTAAG	n/a	n/a	n/a
	n/a		pSK1 rev	TTTATACTGCAATCGGATG	n/a	n/a	n/a
	n/a		A511 CPS fwd-2	GTTTTATGGTATGGCGCATTAGC	n/a	n/a	n/a
Sequencing A511::*nluc*$_{PLY}$	n/a	A511 PLY locus PCR	A511 PLY fwd	TAAGCTAGGAGGAACAACAC	n/a	n/a	n/a
	n/a		A511 PLY fwd-2	AGAGGAAACGGTGTATACG	n/a	n/a	n/a
	n/a		A511 PLY rev	CATTTCCTTTGCTTTCTGC	n/a	n/a	n/a
Sequencing A511::*nluc*$_{CPS}$	n/a	A511 CPS locus PCR	A511 CPS fwd	AACTTGTTCGCGATAACGG	n/a	n/a	n/a
	n/a		A511 CPS fwd-2	GTTTTATGGTATGGCGCATTAGC	n/a	n/a	n/a
	n/a		A511 CPS rev	TGTTTCAGGGTCTACTACACG	n/a	n/a	n/a

n/a = not applicable.

Supplementary Table S3. Synthetic, codon-optimized luciferase DNA strings.

Supplementary Table S3: Synthetic, codon-optimized luciferase DNA strings.

String	Sequence [5'-3']	Size [bp]
rluc	ATCACTA**GAGGAGGTAAATATAT***ATG GGTGGTCGTCGTGTTCGTTGGGAAGTTTAC*<br>*ATCTCTCGTGCTTTATGGTTAACTCGTGAACCAACTGCTTACTGGTTAATCGAAATCA*<br>*ACACTACTCATTACCGTGGTTCTGCTACTATGGCTTCTAAAGTTTACGATCCAGAACA*<br>*ACGTAAACGTATGATCACTGGTCCACAATGGTGGGCTCGTTGTAAACAAATGAACGT*<br>*TTTAGATTCTTTCATCAACTACTACGATTCTGAAAAACATGCTGAAAACGCTGTTATCT*<br>*TCTTACATGGTAACGCTACTTCTTCTTACTTATGGCGTCATGTTGTTCCACATATCGA*<br>*ACCAGTTGCTCGTTGTATCATCCCAGATTTAATCGGTATGGGTAAATCTGGTAAATCT*<br>*GGTAACGGTTCTTACCGTTTATTAGATCATTACAAATACTTAACTGCTTGGTTCGAAT*<br>*TATTAAACTTACCAAAAAAAATCATCTTCGTTGGTCATGATTGGGGTTCTGCTTTAGC*<br>*TTTCCATTACGCTTACGAACATCAAGATCGTATCAAAGCTATCGTTCATATGGAATCT*<br>*GTTGTTGATGTTATCGAATCTTGGATGGGTTGGCCAGATATCGAAGAAGAATTAGCT*<br>*TTAATCAAATCTGAAGAAGGTGAAAAAATGGTTTTAGAAAACAACTTCTTCGTTGAAAC*<br>*TTTATTACCATCTAAAATCATGCGTAAATTAGAACCAGAAGAATTCGCTGCTTACTTAG*<br>*AACCATTCAAAGAAAAAGGTGAAGTTCGTCGTCCAACTTTATCTTGGCCACGTGAAAT*<br>*CCCATTAGTTAAAGGTGGTAAACCAGATGTTGTTCAAATCGTTCGTAACTACAACGCT*<br>*TACTTACGTGCTTCTGATGATTTACCAAAATTATTCATCGAATCTGATCCAGGTTTCTT*<br>*CTCTAACGCTATCGTTGAAGGTGCTAAAAAATTCCCAAACACTGAATTCGTTAAAGTT*<br>*AAAGGTTTACATTTCTTACAAGAAGATGCTCCAGATGAAATGGGTAAATACATCAAATC*<br>*TTTCGTTGAACGTGTTTTAAAAAACGAACAA* ***TAA*** ATCATA	1085
gluc	ATCACTA**GAGGAGGTAAATATAT***ATG GGTGTTAAAGTTTTATTCGCTTTAATCTGTAT*<br>*CGCTGTTGCTGAAGCTAAACCAACTGAAAACAACGAAGATTTCAACATCGTTGCTGTT*<br>*GCTTCTAACTTCGCTACTACTGATTTAGATGCTGATCGTGGTAAATTACCAGGTAAAA*<br>*AATTACCATTAGAAGTTTTAAAAGAAATGGAAGCTAACGCTCGTAAAGCTGGTTGTAC*<br>*TCGTGGCTGTTTAATCTGTTTATCTCATATCAAATGTACTCCAAAAATGAAAAAATTCA*<br>*TCCCAGGTCGTTGTCATACTTACGAAGGTGATAAAGAATCTGCTCAAGGTGGTATCG*<br>*GTGAAGCTATCGTTGATATCCCAGAAATCCCAGGTTTCAAAGATTTAGAACCAATGGA*<br>*ACAATTCATCGCTCAAGTTGATTTATGTGTTGATTGTACTACTGGTTGTTTAAAAGGT*<br>*TTAGCTAACGTTCAATGTTCTGATTTATTAAAAAAATGGTTACCACAACGTTGCGCTAC*<br>*ATTCGCTTCTAAAATACAAGGTCAAGTTGATAAAATCAAAGGTGCTGGTGGTGAT TAA*<br>ATCATA	587
nluc	ATCACTA**GAGGAGGTAAATATAT***ATG GTTTTCACTTTAGAAGATTTCGTTGGTGATTG*<br>*GCGTCAAACTGCTGGTTACAACTTAGATCAAGTTTTAGAACAAGGTGGTGTTTCTTCT*<br>*TTATTCCAAAACTTAGGTGTTTCTGTTACTCCAATCCAACGTATCGTTTTATCTGGTG*<br>*AAAACGGTTTAAAAATCGATATCCATGTTATCATCCCATACGAAGGTTTATCTGGTGA*<br>*TCAAATGGGTCAAATCGAAAAAATCTTCAAAGTTGTTTACCCAGTTGATGATCATCATT*<br>*TCAAAGTTATCTTACATTACGGTACTTTAGTTATCGATGGTGTTACTCCAAACATGATC*<br>*GATTACTTCGGTCGTCCATACGAAGGTATCGCTGTTTTCGATGGTAAAAAAATCACT*<br>*GTTACTGGTACTTTATGGAACGGTAACAAAATCATCGATGAACGTTTAATCAACCCAG*<br>*ATGGTTCTTTATTATTCCGTGTTACTATCAACGGTGTTACTGGTTGGCGTTTATGTGA*<br>*ACGTATCTTAGCT TAA* ATCATA	545

Bold = RBS; italics = coding sequence; red = start and stop codon.

Supplementary Table S4. Experimental set-up for measurement of reporter phage-induced bioluminescence.

Supplementary Table S4: Experimental set-up for measurement of reporter phage-induced bioluminescence.

**Phage**	**Substrate**	***L. monocytogenes* host strain**	**Infection time [min]**	**Culture Temp. [°C]**	**Substrate volume [µL]**	**Culture volume [µL]**
ΦA500::*luxAB* ΔLCR	Nonanal[1)]	WSLC 1042	180	30/20	5	95
ΦA500::*rluc* ΔLCR	Coelenterazine[2)]	WSLC 1042	140	30	50	50
ΦA500::*gluc* ΔLCR	Coelenterazine[2)]	WSLC 1042	140	30	50	50
ΦA500::*nluc* ΔLCR	Furimazine[3)]	WSLC 1042	180	30	50	50
ΦA500 ΔLCR	Coelenterazine[2)]	WSLC 1042	140	30	50	50
	Furimazine[3)]		180	30	50	50
	Nonanal[1)]		180	20	5	95
ΦA511::*luxAB*	Nonanal[1)]	EGDe	180	20	5	95
ΦA511::*nluc*$_{CPS}$	Furimazine[3)]	EGDe	180	30	50	50
ΦA511::*nluc*$_{PLY}$	Furimazine[3)]	EGDe	180	30	50	50
ΦA511	Furimazine[3)]	EGDe	180	30	50	50
	Nonanal[1)]			20	5	95
ΦA006::*nluc* ΔLCR	Furimazine[2)]	EGDe	180	30	50	50
ΦA006 ΔLCR	Furimazine[2)]	EGDe	180	30	50	50

1) 0.35% nonanal in 70% EtOH

2) Renilla Luciferase Assay System (Promega)

3) Nano-Glo® Luciferase Assay System (Promega)
